# The Medicinal Use of the Tomato

**Published:** 1881-11

**Authors:** 


### THE MEDICINAL USE OF THE TOMATO.

Dr. T. K. Griffith, of Hollyrood, Kansas, has found the tomato, eaten raw, or in the form of a fluid extract prepared from the fruit without the use of heat, a very valuable remedy in the treatment of nurse's sore mouth and "canker." In the *Therapeutic Gazette*, for September, 1881, he states that in the summer of 1874 he was treating a case of nurse's sore mouth with chlorate of potassa, carbolic acid and glycerine, iron, quinine, etc. When the earliest ripe tomatoes of the season came into market, the patient saw them, and craved them as food. He directed that she be allowed some; she ate some, and continued to crave them, which craving he allowed her to satisfy. There was a marked improvement in the case from this time; the mouth healed, the appetite returned, and the patient gained strength. Since then he has employed the tomato in a large number of cases, and always with the same success.

